# The role of actin cytoskeleton CFL1 and ADF/cofilin superfamily in inflammatory response

**DOI:** 10.3389/fmolb.2024.1408287

**Published:** 2024-07-24

**Authors:** Jianxiao Xing, Ying Wang, Aihong Peng, Junqin Li, Xuping Niu, Kaiming Zhang

**Affiliations:** ShanXi Key Laboratory of Stem Cells for Immunological Dermatosis, State Key Breeding Laboratory of Stem Cells for Immunological Dermatosis, Taiyuan Central Hospital, Dong San Dao Xiang, Taiyuan, China

**Keywords:** actin cytoskeleton, inflammation, CFL1, ADF/cofilin superfamily, cell migration

## Abstract

Actin remodeling proteins are important in immune diseases and regulate cell cytoskeletal responses. These responses play a pivotal role in maintaining the delicate balance of biological events, protecting against acute or chronic inflammation in a range of diseases. Cofilin (CFL) and actin depolymerization factor (ADF) are potent actin-binding proteins that cut and depolymerize actin filaments to generate actin cytoskeleton dynamics. Although the molecular mechanism by which actin induces actin cytoskeletal reconstitution has been studied for decades, the regulation of actin in the inflammatory process has only recently become apparent. In this paper, the functions of the actin cytoskeleton and ADF/cofilin superfamily members are briefly introduced, and then focus on the role of CFL1 in inflammatory response.

## 1 Introduction

Cytoskeleton is a three-dimensional grid structure composed of interwoven protein fibers, which fills the whole cytoplasmic space and has certain structural connections with the outer cell membrane and the inner nuclear membrane to maintain the unique shape of the cell. Actin filaments, microtubules, and intermediate are the three main cytoskeletal polymers, while stress fiber are bundles of actin filaments. They work together to mediate cell shrinkage, adhesion, and the mechanical force needed for movement to continue reshaping, assembling, and severing (Lambrechts et al., 2004). Actin cytoskeleton is an important part of cell polarization, membrane protrusion, membrane folding, force generation and the formation of membrane protrusions, lamellar pseudopods, membrane folds and adhesive patches. There are many types of actin-binding proteins, such as structural proteins (actin (Pollard and Borisy, 2003), Arp2/3 complex (Martin and Suzanne, 2022), profilin (Davey and Moens, 2020), capping protein (Edwards et al., 2014), and ADF/cofilin (Narita, 2020)), and joint signaling proteins (vinculin (Bays and DeMali, 2017), talin (Klapholz and Brown, 2017), paxillin (Ma and Hammes, 2018), α-actinin (Sjöblom et al., 2008), Focal Adhesion Kinase (Li et al., 2023), Src kinase (Roskoski, 2015)), which are involved in cell migration and proliferation by regulating actin depolymerization (Le Clainche and Carlier, 2008). The signal transduction of these proteins leads to the formation of adhesion sites, and then the signal is transduced to the small GTases of the Rho family, which regulate the organization of the actin cytoskeleton and enable the migration (Biro et al., 2014). Integrin-mediated extracellular matrix (ECM) adhesion called cell-matrix adhesion complexes (CMACs), is dynamically linked to filamentous actin, thereby directly promoting cell migration through ECM adhesion and continuous regulation of actin structure. Inflammatory stimulation often changes the structure of F-actin around the junction in conjunction with apical junctional complex (AJC) breakdown (Ivanov et al., 2010). Overexpression of constitutionally active cofilin accelerates the breakdown of the capsaicin-dependent barrier (Nagumo et al., 2008). Similarly, in ATP-depleted cultured renal epithelial monolayers and experimentally induced *in vivo* rat proximal tubule epithelium, sharp activation of ADF/cofilin coincided with disintegration of the apical F-actin cytoskeleton. ADF/cofilin protein dysfunction may lead to defects in F-actin band assembly around the junction, resulting in damage to AJC structure (Ashworth et al., 2003). In recent years, an increasing number of actin cytoskeleton abnormalities have been associated with immune system diseases (Papa et al., 2020). The dysregulation of actin cytoskeleton in immune cells leads to inflammatory manifestations. ADF/cofilin molecular family role in the inflammatory response has been adequate research, such as macrophages, neutrophils, and so on. Moreover, people have made great progress in many functions such as cofilin mediated actin dynamics, actin nuclear translocation, cell division and migration, and its role in inflammatory response has also been studied (van Rheenen et al., 2009). In this review, we summarize the role of actin cytoskeleton in inflammatory disorders and the function of CFL1 and its role in inflammatory response.

## 2 The role of the actin cytoskeleton in inflammatory response

The actin cytoskeleton is involved in a range of important cellular functions and has a significant impact on many aspects of skin biology. Recent studies have shown that changes in actin cytoskeleton regulation and proteins rearrangement can induce inflammation and immune response [(van Rheenen et al., 2009; Das et al., 2019)]. The remodeling actin proteins include actin nucleators (Arp2/3 complex), actin severing proteins (ADF/cofilin family), actin de-polymerising protein (coronin), nucleation promoting factors (WASp family), actin stabilizing protein and actin severing proteins (Wdr1) (Kopecki et al., 2016). In autoimmune mediated inflammation, WASp-interacting protein (WIP) binding to actin maintains the integration of the actin cytoskeleton and promotes T cell migration (Massaad et al., 2014). Arp2/3 complex-driven spatial patterning of the BCR enhances immune synapse formation, BCR signaling and B cell activation (Bolger-Munro et al., 2019). ADF/cofilin-mediated F-actin remodeling drives macrophage migration, cell polarization, and antigen presentation to T cells (Jönsson et al., 2012). Coronin 1A belongs to an evolutionarily conserved family of actin-binding proteins that regulate actin cytoskeleton-dependent processes. Neutrophil infiltration into gastric mucosa was significantly reduced in coronin 1A^−/−^ mice, resulting in reduced gastric inflammation (Pick et al., 2017). In addition, Wdr1 and cofilin are necessary mediators of immune-cell-specific apoptosis triggered by tecfidera (Poganik et al., 2021). In summary, actin cytoskeleton-mediated F-actin remodeling drives BCR signaling, T cell presentation, neutrophil infiltration, and immune cell specific apoptosis.

In addition, the actin cytoskeleton is regulated by cytokines such as IL6, IL9, IL17, IL-1beta and so on. Cytokines such as IL-9 suppresses the phosphorylation of myosin L chain induced by IFN-γ and IL-17A, which plays a crucial role in regulating actomyosin cytoskeleton and migration potential of human keratinocytes (Das et al., 2019). In human periodontal ligament (hPDL) cells, modulation of the actin cytoskeleton polymerization reaction may activate NF-κB signaling and subsequent expression of leukocyte IL6 and IL8 (Phusuntornsakul et al., 2020). It has also been found that IL-1β reduced long-term potentiation (LTP)-induced F-actin formation, possibly mediated by Src signaling activation of neutral sphingomyelinase (Tong et al., 2018). Cytokines also play an important role in the actin skeleton.

Recruitment of neutrophils is an important component of the immune response in infectious and inflammatory diseases. The intracellular migration and transcellular migration of neutrophils during exudation involve several number of molecules and mechanisms associated with actin cytoskeleton (Glogauer et al., 2021). The release of neutrophils from the bone marrow can recruit cytotoxic mediators at the site of tissue injury, thereby promoting autoimmunity and chronic inflammation (Marzano et al., 2019). The process of neutrophil extracellular traps (NETs) involves a complex multi-step cascade through a closely coordinated sequence of adhesion between endothelial cells in the blood vessel walls (Sprenkeler et al., 2022). Congenital defects of actin rearrangement caused by actin-related protein 2/3 complex subunit 1B (ARPC1B) or megakaryoblastic leukemia 1 (MKL1) deficiency is associated with globally impaired defective cell adhesion and abnormal trafficking of myeloid leukocytes (Sprenkeler et al., 2021). Several endothelial molecules including junctional adhesion molecules, endothelial cell selective adhesion molecules and regulators of endothelial tight junctions have been identified to control transendothelial migration of leukocytes. In addition, the actin cytoskeleton also regulates the function of many inflammatory cells such as macrophages, central granulocytes, dendritic cells, and so on (Lippert et al., 2004; Ishida et al., 2008; Schulte et al., 2011). In the context of autoimmune inflammatory dermatoses and the chemotaxis of macrophages, the interaction between fibroblasts and keratinocytes influences the kinetic function of the actin cytoskeleton during wound healing *in vitro*, including the role of actin in cell aggregation, migration, and contraction (McWhorter et al., 2013). In summary, the actin cytoskeleton participates in the immune response by participating in processes such as B cells, T cells, cytokines, and neutrophil infiltration.

## 3 The function of ADF/cofilin superfamily in inflammatory diseases

ADF/cofilin family members perform mechanistically distinct functions in regulating the actin cytoskeleton. The ADF/cofilin superfamily of remodeling proteins promotes dynamic remodeling of the cytoskeleton, including Cofilins, Destrin, Twinfilin, Drebrin, Glia maturation factor beta (GMF) and coactosin-like protein (COTL1) (Kopecki et al., 2016; Hilton et al., 2018). ADF/Cofilin protein binds to F-actin and induces fragmentation and depolymerization (Wioland et al., 2017). Twinfilins increase the depolymerization rate of the barbed ends and tips of actin filaments. Destrin binds specifically to F-actin and affects actin organization *in vivo*, but has no known effect on actin filament dynamics *in vitro*. GMF binds with high affinity to the actin-associated protein (Arp) 2/3 complex but not to actin and catalyzes actin filament debranching to promote network remodeling and turnover (Gandhi et al., 2010).

### 3.1 Destrin (DSTN)

Destrin (DSTN) is widely present in various tissues. Destrin binds to filamentous actin subunits, enhances the subunit dissociation rate and promotes filamentous fragmentation, which promotes tumor cell migration and invasion (Zhang et al., 2020). DSTN deletion has different effects on spontaneous angiogenesis and lymphangiogenesis in normal vascularized cornea (Cursiefen et al., 2005). Cellular hyperproliferation, inflammation, and angiogenesis are biological processes at the heart of the pathogenesis of corneal diseases as well as other conditions such as chronic inflammatory diseases. Corneal disease 1 (Dstn) mice were found to be homozygous for the spontaneous null allele of the desmin (Dstn) gene, and these mice exhibited corneal abnormalities, including epithelial hyperproliferation, stromal inflammation, and neovascularization (Verdoni et al., 2010). The inflammatory chemokine CXCL5 is ectopically expressed in corneal epithelial cells of DSTN mice, and targeting the receptor of this chemokine inhibits neutrophil recruitment (Verdoni et al., 2008).

### 3.2 Twinfilins

Twinfilins regulate actin dynamics and have synergistic effects with Arp2/3, cortical proteins and other actin, participating in the formation of lamopodia, filopodia and other cell processes (Vartiainen et al., 2003). TWF-1 (Twinfilin-1) plays a certain role in entosis (invasive cell death: cells enter other cells and then die) and cell migration of mammalian cells (Poukkula et al., 2011), while TWF-2a plays a role in neuronal morphogenesis. TWF1 was correlated with immune cell infiltration in the tumor stroma. The relationship between TWF1 and immune checkpoints programmed cell death 1(PD1) and cytotoxic T lymphocyte–associated antigen-4 (CTLA4) and sensitivity to immunotherapy was further analyzed, illustrating that in CTLA4^−^ PD1^−^ and CTLA4^+^ PD1^−^ patients, the immune scores (The Cancer Imaging Archive database (https://tcia.at/)) were significantly higher in patients with lowly expressed TWF1 than those in patients with highly expressed TWF1. This provided support for TWF1’s significance in lung adenocarcinoma (LUAD) immunity (Zhai et al., 2023).

### 3.3 Drebrin 1 (DBN1)

DBN1 is an actin-associating protein enriched at the cell-cell junction, forming a specific microfilament anchorage system in polarized epithelial cells (Peitsch et al., 1999). In neuronal cells, DBN1 couples dynamic microtubules to F-actin in growth cone filopodia and organizes F-actin in dendritic spines, having a critical role in neuritogenesis and synaptic plasticity (Ivanov et al., 2009). In Arctigenin (ATG) treated podocytes, DBN1 phosphorylation at T335 is reduced by activation of protein phosphatase 2 A (PP2A), which is essential for the interaction of DBN1 with F-actin and the actin cytoskeleton in podocytes. The decrease in DBN1 in human diabetic glomeruli may be due to podocyte loss during late diabetic kidney disease (DKD) rather than a decrease in the DBN1 gene itself (Zhong et al., 2019). The profibrotic role of drebrin through actin cytoskeleton formation and the regulation of drebrin on collagen triple helix repeat containing 1 (Cthrc1) expression in hepatic myofibroblasts (Hironaka et al., 2023). In addition, in Alzheimer’s disease (AD), the expression of DBN1 is increased, while the inflammatory response is reduced, resulting in the improvement of memory (Espinosa-Jiménez et al., 2023).

### 3.4 Glia maturation factor beta (GMF)

Glia maturation factor (GMF) is a 17-kDa pro-inflammatory protein that is present in glial cells and some neurons. In Alzheimer’s disease (AD), GMF expression is reduced and improves neuroinflammatory responses by inhibiting pro-inflammatory cytokines (TNF-α, Il-1β, and IL-1) (Ahmed et al., 2017). Under certain circumstances, such as Aβ deposition and oxidative stress, GMF can be overexpressed and play its lethal role in the progression of neurodegenerative diseases such as AD and Parkinson’s disease (PD) (Ahmed et al., 2021). In GMF-KO mice, pro-inflammatory cytokines (TNF-α and IL-6) were gradually decreased and anti-inflammatory cytokines (IL-4 and IL-10) were gradually up-regulated, promoting the shift of microglia to a more predominant anti-inflammatory (M2) phenotype. GMF-KO mice showed significant improvements in motor ability, memory, and cognition (Ahmed et al., 2020).

### 3.5 Cofilins

Cofilins, members of the extensively studied ADF/cofilin superfamily, as we all know, regulate actin kinetics by cutting actin filaments to catalyze actin depolymerization or polymerization (Shishkin et al., 2016). The concentration of cofilins is important for the assembly and disassembly of actin filaments, and the ratio of Globular-actin (G-actin) to filamentous (F-actin) and a large number of protein factors also play a role in maintenance of cell motility, cell shape, and polarity to regulation of transcription (Andrianantoandro and Pollard, 2006). At low concentrations of ADF/cofilin, actin filaments are cleaved and depolymerized, while high concentrations of cofilin promoted actin polymerization and nucleation (Wang et al., 2015). Although we have recognized some functions of CFL1, it is more important to apply it to the treatment and pathogenesis of diseases. Nuclear factor-κB (NF-κB), composed of a family of transcription factors, regulates the expression of inflammatory genes. Cofilin has been involved in hypertension-induced kidney damage by regulating NF-κB in renal tubular epithelial cells. Under the stimulation of LPS, cofilin is activated and mediates F-actin disassembly, which enhances the production of cytokines in T lymphocytes by activating nuclear factor of activated T cells (NFAT) thereby activating NF-kB pathway (Alhadidi and Shah, 2018). Cofilin induces microglia activation by activating the NF-kB pathway and the JNK stress kinase pathway, which can promote the expression of inflammatory mediators and cause inflammatory diseases (Nennig and Schank, 2017). Cannabinoid Receptor 2 (CB2) antagonist AM630 reversed tetrahydrocannabinol (THC) induced modulation of cofilin-1 expression in MG-63 cells, and cofilin-1 overexpression abolished THC-induced cellular anti-inflammatory effects, suggesting that cofilin-1 may mediate THC-induced anti-inflammatory effects through CB2 receptors (Yang et al., 2015). Because of the role of cofilins in inflammation and immune diseases, they can potentially be used as a biomarker in the diagnosis and evaluation of the application of disease activity (Hou et al., 2021).

### 3.6 Coactosin like protein 1 (COTL1)

Coactosin like protein 1 (COTL1) prevents actin cofilin-mediated depolymerization and thus promotes lamellipodia formation (Shao et al., 2020). COTL1 is tightly associated with F-actin bundles at the growth cone, and overexpression of actinin promotes lamellipodia expansion and growth cone extension. The expression of COTL1, a component of senescence associated secretory phenotype (SASP) secreted by senescent L02 hepatocytes in response to Cr(VI), was significantly up-regulated, suggesting that SASP may promote tumor development through chronic inflammatory response (Ma et al., 2021). In addition, COTL1 interacts with arachidonate 5-lipoxygenase (ALOX5) and participates in the biological process of immune cell activation and the functional pathway of immune response (Zhao et al., 2023).

As a whole, ADF/cofilin superfamily proteins play a multifaceted role in cells. Since they are involved in immune of mammalian cells, they can also be implicated in various pathological processes. The study of the possible contribution of these proteins to immune is an important task of molecular.

## 4 Structure and function of the ADF/cofilin superfamily member: cofilin-1 (CFL1)

Cofilin is a member of the ADF/cofilin superfamily that is important for the regulation of actin cytoskeleton dynamics. Cofilin is a conserved protein with a molecular weight of 19 kDa. It was first discovered in the chicken brain and binds to actin monomers in filaments at a 1:1 molar ratio (Hild et al., 2014). It belongs to the actin depolymerization factor (ADF)/cofilin family, which consists of three isoforms (cofilin-1, cofilin-2 and ADF) in mammals. The structure of cofilin is relatively conserved, mainly consistent of the actin-depolymerizing factor homology (ADF-H) domain, which interacts with Arp2/3 complexes, Globular-actin (G-actin) and filamentous (F-actin) (Moon and Drubin, 1995). The dimensional structure of cofilin has two pairs of α-helices, sandwiched by a mixed β-fold (Vartiainen et al., 2002). All eukaryotic cells contain an cofilin to perform their biological functions (Ono et al., 1994). In mammalian cells, there are two subtypes of cofilin. Non-muscle type (NM-type) actin (cofilin-1), which is mainly present in a variety of non-muscle tissues. The other encoding M-type (muscle type, cofilin-2) actin, is mainly expressed in muscle cells and can also be expressed in tissues such as heart and skeletal muscle (Gillett et al., 1996).

**FIGURE 1:**
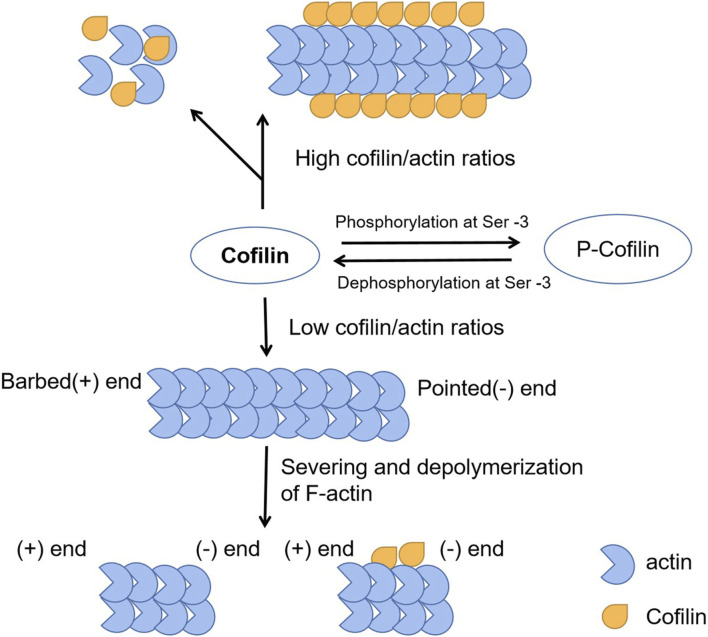
The function of CFL1 to actin filaments. The basic function of CFL1 is to accelerate the movement of actin filaments by depolymerizing and severing them.

The basic function of CFL1 is to accelerate the movement of actin filaments by depolymerizing and severing them (Figure 1). The latter enhances actin filament polymerization and promotes physiological behaviors such as cell motility (Kanellos and Frame, 2016). The depolymerization activity of CFL1 promotes depolymerization from the tip of actin filaments (Maciver and Hussey, 2002). The tip of the actin filament contains the actin subunit bound by the CFL1. The binding of CFL1 to actin subunit opens the nucleotide binding gap on filament, resulting in an increase in the average distance of neighboring actin subunits on the long axis filament and weakening the interaction between actin subunits (Fan et al., 2013). Low concentration of actin filament protein can cut off the depolymerization of actin filaments and promoting, and high levels of actin filament protein, promote actin nucleation and aggregation. Actin cofilin nucleates actin polymerization directly or indirectly in a concentration dependent manner (Bravo-Cordero et al., 2013). It can promote F-actin assembly by stabilizing preexisting filaments and nucleating new filaments at high concentrations, whereas it can promote F-actin disassembly by accelerating the dissociation of monomeric actin from the negative end of actin filaments and cutting F-actin at lower concentrations (Wang et al., 2020). Actin filament severing can result in either net assembly or disassembly of F-actin, depending on the activity of actin polymerizing proteins and the local g-actin concentration. To sum up, ADF/cofilins play an essential role in the controlling of actin dynamics. They have a dual effect on actin filaments and may contribute to cellular contractility through both the local actin depolymerization and the formation of stress fibers, and therefore they are important for morphogenesis and development.

In addition, it has been shown that cofilin promotes the binding of tropomyosin to motile actin networks generated by the Arp2/3 complex and capping protein (Hsiao et al., 2015). The Arp2/3 protein complex consists of seven subunit complexes capable of binding to actin and providing actin branch nucleation and formation (Dos et al., 2003). The formation of actin branches is essential for cell movement. CFL1 and Arp2/3 work together to produce free barb terminus for actin polymerization (Tania et al., 2013). At the same time, CFL1 promotes dissociation of old actin offshoots and reduces the Arp2/3 complex action on the actin filaments (Dumpich et al., 2015). The Ras-related C3 botulinum toxin substrate 1 (Rac1)-WASP-family verprolin-homologous protein-2 (WAVE2)-actin-related protein 2/3 (Arp2/3) signaling pathway increases radiation resistance in U251 human glioma cells through CFL1 (Zhou et al., 2016).

The activity of cofilin is regulated by various mechanisms. Cofilin activity is regulated by pH, phosphatidylinositol, protein kinases and phosphatases, and a number of other proteins. It is well known that the F-actin binding and depolymerization activity of cofilin depends on pH. Yonezawa et al., *in vitro*, in model in the system containing F-actin, when pH < 7.3, monomer actin (G-actin) concentration is lower than 1 µM. However, when the pH > 7.3, G-actin protein concentration and adding wire cut is proportional to the concentration increased, until F-depolymerization of actin completely (Bernstein et al., 2000). The initial activation of cofilin in breast tumors is dependent on plcγ, whereas cofilin activation in neutrophils is also dependent on dephosphorylation promoted through the Rho-family small GTPase Rac2 signaling. This may use different starting points in the cofilin activity cycle, but the output signal is the same local cofilin activity initiates local actin polymerization, membrane extrusion, directed migration, and even chemotaxis (Zhou et al., 2016). CFL1 can directly bind to phosphatidylinositol 4,5-bisphosphate (PIP2), resulting in inhibition of actin binding. Reduced cofilin expression in leukocytes is associated with abnormal chemotaxis (Bamburg and Wiggan, 2002). In neurons, cofilin controls axon elongation and regeneration and serum levels are significantly higher in patient with Alzheimer’s disease (Sun et al., 2019). Cyclase-associated protein 1 (CAP1) and actin-interacting protein 1 (AIP1) promote the disassembly of actin cofilin bound actin filaments. The cellular redox state also modulates the activity of ADF/cofilins (Nomura et al., 2016). Thus, cofilin play a multifaceted role in cells involved in different pathological processes.

Cofilin also regulate cell proliferation, promoting cofilin-mediated actin cytoskeletal remodeling (Hawkins et al., 2022). A study showed that cell proliferation in the epithelial monolayer is profoundly influenced by mechanical regulation of transcription factor YAP1 (YES-related protein 1), TAZ1 (transporter-related zinc finger protein 1) and cytoskeletal checkpoints, which are enforced by the actin cap and cut-off proteins cofilin, CapZ and Gelsolin (Aragona et al., 2013). YAP and TAZ activities are key factors for tissue-specific progenitor cell expansion and proliferation throughout organ growth, tissue renewal and regeneration, and are linked to a variety of diseases, including cancers (Tang et al., 2022). The crosstalk between YAP and TAZ and the actin cytoskeleton plays a regulatory role in cutaneous melanoma (Brouwer et al., 2021). The reduced activity of cofilin in cells of uveal melanomas promotes actin cytoskeleton stability and YAP activation. Taken together, a line of evidence suggests that cell can sense the structure of the microenvironment and respond to mechanical signals through cofilin dependent remodeling of the actin cytoskeleton, triggering responses critical to cell fate and tissue homeostasis.

Alternatively, the role of cofilin during reproductive development has been described. This was demonstrated by embryonic lethality in cofilin-1 KO mice. Nonmuscular cofilin was identified as a component of the tubular-medulla complex in rat setoli cells and as a key activator of capacitation and acrosome reaction in human sperm (Xu et al., 2023). The testis-specific LIMK2 isoform (tLIMK2) is specifically expressed at the meiotic stage of spermatogenic cell differentiation, suggesting its contribution to spermatogenesis (Park et al., 2021). Abnormal F-actin dynamics also contribute to acrosome dysplasia during sperm head shaping, resulting in sperm head and acrosome malformations (Zhu et al., 2023). Enhanced cofilin phosphorylation and disorganized F-actin were observed in slingshot phosphatase 2 (SSH2) KO testes, along with impaired trafficking of anterior acrosomal vesicles. Cofilin/ADF is also an important regulator of actin cell motility during *drosophila* development.

### 4.1 Function of CFL1 in the nucleus

**FIGURE 2:**
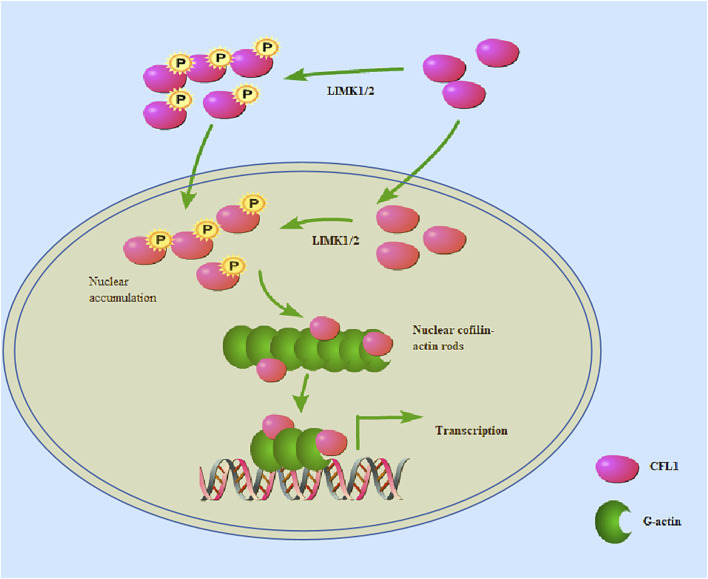
CFL1 can be phosphorylated by LIMK1/2 in either the nucleus or the cytoplasm. CFL1 is phosphorylated by LIMK1/2 in the nucleus. When phosphorylated CFL1 is inactivated, actin filaments are depolymerized and nuclear actin rods are formed. LIMK1/2 in the nucleus phosphorylates cofilin, and cause actin rearrangement. Nevertheless, some researchers have proposed that phosphorylated cofilin can transit from the cytoplasm to the nucleus.

Cofilin plays an important role in the polymerization of actin in the nucleus. The protein sequence of CFL1 contains nuclear localization signal (NLS), providing CFL1 a special ability to bind to and carry actin depolymerize into the nucleus. The NLS fragment of CFL1 functions upon link to non-nuclear proteins in muscle ducts (Pendleton et al., 2003). Cofilin is known to be actively imported into the nucleus via either the classical import protein or the nuclear transporter α/β (α/β) -dependent NLS (21-RKSSTPEEVKKRKK-34) (Iida et al., 1992). When a synthetic peptide containing NLS is injected into the cell, the synthetic peptide rapidly accumulates in the nucleus. Since actin lacks a NLS, ADF and cofilin may serve as chaperones to transport actin into the nucleus under conditions that induce rod formation (Munsie et al., 2012). Nuclear actin is also important for gene transcription by binding to RNA polymerase I, promoting RNA processing and exporting mRNA to the cytoplasm (Philimonenko et al., 2004). Under the stimulation of mechanical injury or inflammatory factors (EGF, IL-4, IL-8, etc.), cofilin is an accessory molecule that transports actin to the nucleus for gene transcriptional expression. Cofilin can promote the release of nuclear transcriptional cofactors, such as glucocorticoid receptor (GR) and serum response factor (SRF) -related cofactors (Minamide et al., 2000). Cofilin transmits the protein kinase cascade to the actin cytoskeleton through site-specific phosphorylation at residue serine3 (Hamill et al., 2016). When LIM kinase phosphorylation is inactivated, the serine residue at position 3 of cofilin is phosphorylated and fails to activate downstream nuclear localization signals, thereby blocking G-actin accumulation in the nucleus. The substitution of Ser-3 in the cofilin N-terminus by aspartate (Asp) causes steric conflicts that dissociate and reorient the actin cofilin N-terminus from actin, thereby reducing the total number of actin-actin interface contacts and weakening the intrinsic binding affinity of actin cofilin (Elam et al., 2017). Cofilin/actin rod formation has also been found in the nucleus and cytoplasm of neurodegenerative diseases, including Alzheimer’s disease (AD) and Parkinsonism (Maloney and Bamburg, 2007). Regulation of the actin cytoskeleton by cofilin is considered to be important in pathological studies of aging or stress related diseases such as neurodegenerative diseases (Bamburg et al., 2010). The dephosphorylated form of cofilin is essential for entry into the nucleus (Li et al., 2014). A recent report showed that phosphorylated cofilin can transit from the cytoplasm to the nucleus in the laminar formation of the chicken optic tectum, indicating that phospho-cofilin plays a role in neural development biology (Yang and Mizuno, 1999). On the other hand, CFL1 is phosphorylated by LIMK1 and LIMK2 in the nucleus (Slee and Lowe-Krentz, 2013). When CFL1 is inactivated, actin filaments are depolymerized and nuclear actin rods are formed (Figure 2). Actin rods binds to chromatin modification complexes associated with INO80 and is essential for chromatin remodeling (Kapoor and Shen, 2014).

Nuclear actin is also important for gene transcription by binding to RNA polymerase, which facilitates RNA processing and exports mRNA to the cytoplasm (Elam et al., 2017). In addition, p57kip2, a cyclin dependent kinase (CDK) inhibitor, binds to LIMK1 and then translocate to the nucleus to reorganize actin fibers (Yokoo et al., 2003). These mechanisms account for the activated LIMK1/2 on the formation of phospho-CFL1 in the nucleus. However, whether this biological behavior of CFL1 forms specific actin bar structures within the nucleus or phosphorylated CFL1 causes actin rearrangement warrants further investigation. In summary, cytoplasmic to nuclear conversion of both phosphorylated and non-phosphorylated actin filagins may play an important role in various biological processes, and therefore deserves further study.

### 4.2 The function of CFL1 in maintaining cell-cell contacts

CFL1 also plays a role in maintaining cell-cell contacts. The tight regulation between cell connectivity and migration is fundamental during homeostasis and in disease including metastatic cancers. However, the mechanisms regulating junctional cell attachment and cell migration are not fully understood yet. In keratinocytes, the rupture of junctional complex followed by changes in actin-based membrane dynamics increases cell motility (Kanellos et al., 2015). Consistently, we demonstrated that cofilin-1 regulation of actin dynamics can control malignant behaviors, modulating junctional complex organization during EMT in colon cancer cells (Sousa-Squiavinato et al., 2019). E-cadherin is a calcium-dependent adhesion receptor, which is essential in the process of cell adhesion and can dynamically regulate and stabilize intercellular adhesion (Braga, 2016). F-actin is very unstable in siCofilin-1 cells under TGF-β induction, but E-cadherin and claudin-3 restore intercellular adhesion. The expression of CFL1 (phosphorylated or non phosphorylated) and its regulator LIMK is in a state of homeostasis and is essential for changes in actin cytoskeleton dynamics (Wang et al., 2006). In addition, LIMK2 can increase cortical actin depolymerization/severing by increasing the activity of CFL1 when cells are in contact with each other (Ohashi et al., 2011). LIMKs and CFL1 plays a key role in the deceleration and acceleration of retrograde actin. In psoriatic keratinocytes, LIMK1 is involved in coordinating the dynamics of microtubules and actin, and LIMK1 expression in the granular layer mediates cell compaction by negatively regulating cofilin (Honma et al., 2006). CFL1 can act as a transition between phosphorylated and dephosphorylated states in different subcellular regions (Shankar and Nabi, 2015).

**FIGURE 3:**
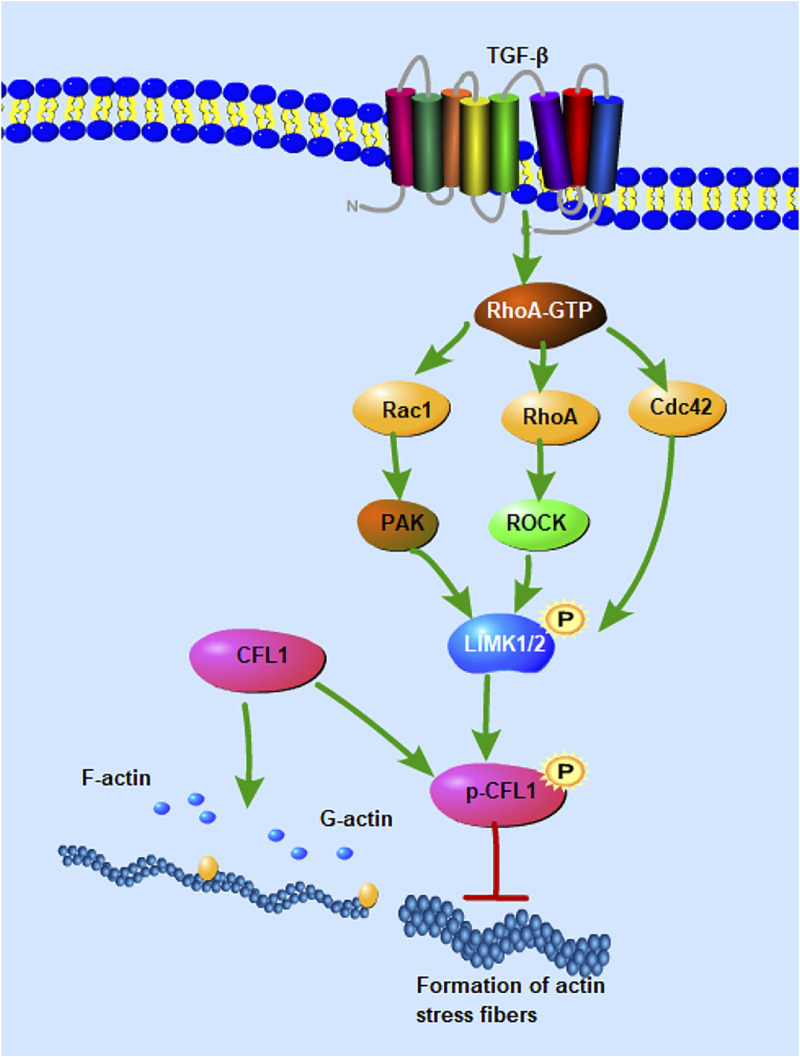
Signaling pathways involved in the regulation of CFL1 activity. CFL1 regulates F-actin depolymerization and nucleation activity through phosphorylation and dephosphorylation. Rho and Cdc42 induce actin cytoskeletal reorganization by mediating LIMK1/2 activity. RhoA activates Rho-associated protein kinase (ROCK), phosphorylates and activates LIM kinase (LIMK), and activated LIMK phosphorylates and regulates the activity of cofilin. Rac1 binds and activates P21-activated kinase (PAK), which in turn phosphorylates and activates LIMK.

Cofilin activation/inactivation are modulated by changes in balance of kinases, phosphatases and other cofilin upstream regulatory proteins. Rho GTPase signaling is able to regulate the actin cytoskeleton. Rho GTPases link TGF membrane receptors to the regulation of adhesion complexes (Figure 3), actin, and gene transcription to facilitate a coordinated transformation of cellular behavior (Hall, 2005). Rho GTPases are composed of many molecules, such as the small GTPase Ras homolog gene family member A (RhoA), the small GTPase Ras-related C3 botulinum toxin substrate 1 (Rac1), and cell division control protein 42 (Cdc42). The N-terminus of RhoA activated Rho-associated protein kinase (ROCK) contains catalytic kinase domains near the curly helix region, including the rho binding domain (RBD) and the PH domain. Active RhoA binds to the RBD of ROCK and activates it by disrupting the auto-inhibitory activity of the N- and C-terminal binding (Bros et al., 2019). ROCK can phosphorylate and activate LIM kinase (LIMK), while the activated LIMK phosphorylates and inhibits the activity of cofilin. Rac1 binds to and activates p21-activated kinases (PAKs), which in turn phosphorylate and activate LIMK (Kassianidou et al., 2017). CFL1 has been shown to be phosphorylated and inactivated by LIM kinases (LIMK1, LIMK2) and testicular protein kinase (TESK1). In contrast, actin cofilin can be dephosphorylated and activated by slingslin phosphatases (SSH1, SSH2, SSH3), protein phosphatases 1 and 2A (PP1, PP2A), and chronophin (CIN) (Aragona et al., 2013). TESK1 can phosphorylate cofilin at the Ser-3 site *in vivo* and *in vitro*, thereby affecting actin organization. The N-terminal domain of SSH1 interacts with actin cofilin, where the Cys-393 residue of SSH1 is essential for removing the phosphate group on Ser-3 of actin cofilin. SSH1 also dephosphorylates LIMK1 and attenuates LIMK1’s enzymatic activity on actin cofilin (Namme et al., 2021). Phosphorylation of Rho-associated kinases (ROCK1 and ROCK2) activates non-muscle myosin II motors and promotes actin filament assembly by mammalian diaphanous-related (mDia) formin proteins (Sahai and Marshall, 2002). The RhoA-ROCK pathway plays a direct role in regulating the delicate balance between cellular responses to substrate stiffness and migration, which are crucial for processes such as neutrophil migration, wound healing, endothelial and epithelial barrier function, as well as human keratinocyte proliferation and differentiation (Lock and Hotchin, 2009). Additionally, Rho-dependent actin polymerization can modulate the abundance of monomeric G-actin pools in both the cytoplasm and nucleus, thereby influencing transcriptional regulation (Kassianidou et al., 2017). Therefore, CFL1 regulates intercellular connectivity by modulating actin dynamics, and RhoA-ROCK signaling regulates protein distribution by regulating actin remodeling.

## 5 CFL1 regulation of the inflammatory response

CFL1 play a crucial role in B cell, T cell infiltration, cytokine, and NF-κB signaling pathways. Cofilin activity is regulated by phosphorylation of serine 3 (S3), which prevents Cofilin from binding to actin filaments. Lim-domain kinases (LIMK) 1 and 2 are widely expressed kinases that phosphorylate kinetin filagins on S3. LIMK is activated by phosphorylation of ROCK, a downstream target of Rho GTPase, or P21-activated kinase (PAK), an effector of Rac and Cdc42 GTPase. The Rho-ROCK-LIMK pathway regulates the formation and function of immune synapses in T cells by either expressing constitutively active ROCK or inhibiting actin filamin activity by depleting actin filamin with siRNA (Glotfelty et al., 2023).

### 5.1 CFL1 regulates immune responses in T and B cells

CFL1 regulates the immune response of T and B cells. Studies have shown that patients with primary sjogren’s syndrome (pSS) exhibit autoantibodies against actin-1. pSS is an autoimmune disease characterized by damage to the salivary and lacrimal glands, resulting in dry eyes and dry mouth, and additional exocrine glands and other tissues may also be involved in the disease. Pathological studies have shown that T, B, and NK cells carry out progressive lymphocytic infiltration of the affected glands (Campos et al., 2009). In addition, in a mouse model of imiquimote-induced psoriasis, cofilin-deficient mice lacked peripheral αβ T cells and exhibited severe thymus atrophy (Seeland et al., 2018).

### 5.2 CFL1 regulates inflammatory responses by cytokines

The inflammatory response is characterized by the coordinated activation of various signaling pathways that regulate the expression of pro-inflammatory and anti-inflammatory mediators in recruited leukocytes in the blood. Currently, most of our knowledge of signaling in inflammatory is gained from the research on members of the IL-1 and TNF receptor family members and Toll-like receptors (TLRs). Mice with lung inflammation caused by lung cell shedding carry a large amount of IL-1 and TNF-α. Blocking the phosphorylation of cofilin can not only inhibit the formation of lung microvesicles, but also reduce the lung injury of mice (Zhang et al., 2018). In studies of acute ischemic kidney injury, microvascular endothelial cells (MS1) exposed to a mixture of IL-1α, IL-6, and TNF-α cytokines undergo a significant, time-dependent redistribution of the actin cytoskeleton and form long, dense F-actin basal stress fibers within minutes. Increased phosphorylated cofilin and RhoA activation further suggest that cytokines signal through the RhoA-ROCK pathway to influence actin dynamics (Tur-Gracia and Martinez-Quiles, 2021). TLR-activated macrophages strongly induce synthesis and secretion of Wnt5A, which has been determined as a TLR-activated chemokine secreted by human macrophages, playing a key role in the autocrine regulation of inflammatory response (Skaria and Schoedon, 2017). Wnt5A regulates cytoskeletal remodeling and barrier function by Ryk receptor and ROCK, targeting LIMK2 and CFL1 involved in actin polymerization (Skaria et al., 2017). Interleukin-1β (IL-1β) stimulation in human corneal endothelial cells (CECs) temporarily induces Wnt5a via NF-κB. This leads to activation of Cdc42 and subsequent inhibition of RhoA, which leads to cofilin dephosphorylation which enhances cell migration (Lee and Heur, 2014). Interleukin 4 (IL-4) is a potent Th2 cytokine that is known to impair endothelial barrier function. IL-4 increases the permeability of the HCAEC monolayer and impairs barrier function. Silencing Wnt5A significantly reduced the permeability of the HCAEC monolayer and improved its barrier function after IL-4 treatment. Wnt5A, as an effector, can mediate actin cytoskeleton remodeling in IL-4-activated human coronary artery endothelial cells (HCAEC) by regulating LIM kinase (LIMK) and cofilin (Skaria et al., 2016). The above studies suggest that activation of CFL1 under the stimulation of cytokines leads to the remodeling of actin and thus plays a role in inflammatory diseases.

### 5.3 CFL1 regulates inflammatory responses by activating the NF-κB signaling pathway

Disruption of actin cytoskeleton results in activation of NF-kB and production of inflammatory mediators in intestinal epithelial cells (Németh et al., 2004). NF-κB activation is balanced by inhibitory signals, mainly including stimuli that elevate intracellular levels of the second messenger 3′-5′ cyclic adenosine phosphate (cAMP) (Gerlo et al., 2011). cAMP activation of PKA does not only inhibit TNF-α induced NF-κB dependent reporter gene expression, but also reduces NF-κB dependent expression of adhesion molecules and chemokines (Aizawa et al., 2003; Aoki et al., 2010). TNF-α can induce the increase of NF-κB expression, which is inhibited by the high expression of cAMP and nuclear actin monomer. Elevated cAMP and nuclear actin monomers inhibits RhoGTPases, thereby reducing cytoplasmic actin polymerization and actin stress fiber formation, resulting in inhibition of actin dependent transcription co-factors such as YAP/TAZ (Bond et al., 2008).

Activation of NF-κB is induced by inhibition of the degradation of IκBα and IκBβ proteins and nuclear translocation of their subunits p65, p50,cRel, RelB and p52 (Hayden and Ghosh, 2008; Rahman and Fazal, 2011). Among the many subunits, the NF-κB p65 subunit has an activation domain at the C-terminus for transcriptional activity (O Shea and Perkins, 2008). Dynamic changes in the actin cytoskeleton require translocation of RelA/p65 (a subunit of NF-κB) to the nucleus, resulting in an inflammatory response. The formation of stress fibers increases with the loss of CFL1 and inhibits the nuclear accumulation of RelA/p65 (O Shea and Perkins, 2008). Reduction in nuclear actin monomer levels increases RelA/p65 protein levels and NF-κB activity in cells with elevated cAMP signaling.

In recent years, small molecule inhibitors of cofilin have been developed successively. Bahader et al. (Bahader et al., 2023) found that when CI, a small molecule inhibitor of cofilin, acts on human neuroblastoma (SH-SY5Y) and microglia (HMC3), the expression of cofilin is significantly decreased, and the release of pro-inflammatory mediators is reduced, preventing the accumulation of ROS induced by H_2_O_2_ and neuronal cytotoxicity. Two other inhibitors, SZ-3 and SK-1–32, reduce NO and prevent neurotoxicity to prevent microglia from LPS-induced inflammation, while SK-1–32 also reduces the expression of TNF-α and nuclear factor κB (NF-κB) (Alaqel et al., 2022).

## 6 Summary

The actin cytoskeleton is a collection of actin filaments and their accessory and regulatory proteins. The activity of the actin cytoskeleton supports the vast majority of motor events in cells. A large number of accessory proteins control the assembly of the common pool of actin, as well as spatial organization, subcellular localization, and interactions of filaments with other structures. The cytoskeleton is intrinsically involved in all aspects of inflammation, including inflammatory cell migration, adhesion to affected areas, and regulation of cytokine production and cell signaling. Recently, the regulation of ADF/cofilin molecular family functions and the interaction with microfilament actin have been studied in more and more detail. Great progress has been made in understanding the structural function and biological role of cofilin, and its role in inflammatory responses has been well studied. Kinase (LIMK1) can activate cofilin, and LIMK1 has been widely studied as a regulator of cofilin-mediated cell motility and inflammation. The release of cofilin increases the breaking of actin filaments. CFL1 is involved in many aspects of inflammation, including the migration of leukocytes, lymphocytes, etc., and the regulation of the production of cytokines TNF, IL1, IL6, etc. CFL1 causes the accumulation of stress fiber and nuclear translocation of NF-κB p65, which promotes the development of inflammation. Cofilin is an inflammatory mediator that broadly participates in many diseases, and can serve as a useful biomarker for diagnosis of inflammatory diseases, and a target in the management of inflammatory disorders.

## 7 Future directions

In summary, the cytoskeleton plays a multi-factorial role in the migration of inflammatory cells, adhesion to the affected area, cytokine production and cell signaling regulation. The regulation of cofilin phosphorylation appears to be particularly important in the inflammatory response. However, there are several key issues that need to be addressed further. First, while cofilin regulates actin polymerization, how changes in actin lead to inflammation remains unclear. Second, the relationship between the accumulation of inflammation and phosphorylation/dephosphorylation of cofilin needs to be further characterized.
